# Recent Advances on Nanomaterials to COVID‐19 Management: A Systematic Review on Antiviral/Virucidal Agents and Mechanisms of SARS‐CoV‐2 Inhibition/Inactivation

**DOI:** 10.1002/gch2.202000115

**Published:** 2021-02-22

**Authors:** Anna Paula A. Carvalho, Carlos A. Conte‐Junior

**Affiliations:** ^1^ COVID‐19 Research Group Technological Development Support Laboratory (LADETEC) Department of Biochemistry Federal University of Rio de Janeiro (UFRJ) UFRJ Rio de Janeiro 21941‐909 Brazil; ^2^ COVID‐19 Research Group Laboratory of Advanced Analysis in Biochemistry and Molecular Biology (LAABBM) Institute of Chemistry (IQ) Federal University of Rio de Janeiro (UFRJ) UFRJ Rio de Janeiro 21941‐909 Brazil; ^3^ Graduate Program in Chemistry (PGQu) Institute of Chemistry (IQ) Federal University of Rio de Janeiro (UFRJ) Rio de Janeiro 21941‐909 Brazil; ^4^ Graduate Program in Food Science (PPGCAL) Institute of Chemistry (IQ) Federal University of Rio de Janeiro (UFRJ) Rio de Janeiro 21941‐909 Brazil; ^5^ Nanotechnology Network Carlos Chagas Filho Research Support Foundation of the State of Rio de Janeiro (FAPERJ) Rio de Janeiro 20020‐000 Brazil; ^6^ Graduate Program in Veterinary Hygiene (PPGHV) Faculty of Veterinary Medicine Fluminense Federal University (UFF) Niterói 24230‐340 Brazil; ^7^ Graduate Program in Sanitary Surveillance (PPGVS) National Institute of Health Quality Control (INCQS) Oswaldo Cruz Foundation (FIOCRUZ) Rio de Janeiro 21040‐900 Brazil

**Keywords:** bioactive compounds, cell mechanisms, nanomedicine, polymeric nanoparticles, self‐cleaning surfaces

## Abstract

The current pandemic of coronavirus disease 2019 (COVID‐19) is recognized as a public health emergency of worldwide concern. Nanomaterials can be effectively used to detect, capture/inactivate or inhibit coronavirus cell entry/replication in the human host cell, preventing infection. Their potential for nanovaccines, immunoengineering, diagnosis, repurposing medication, and disinfectant surfaces targeting the novel coronavirus (SARS‐CoV‐2) is highlighted. In this systematic review the aim is to present an unbiased view of which and how nanomaterials can reduce the spread of COVID‐19. Herein, the focus is on SARS‐CoV‐2, analyzing 46 articles retrieved before December 31, 2020. The interface between nanomaterials is described, and the main mechanisms to inhibit SARS‐CoV‐2 pathogenesis and viral inactivation are also discussed. Nanocarbons, biopolymeric, copper, and silver nanoparticles are potential antiviral and virucidal agents toward self‐cleaning and reusable filter media and surfaces (e.g., facial masks), drug administration, vaccines, and immunodiagnostic assays. Trends in toxicology research and safety tests can help fill the main gaps in the literature and overcome health surveillance's challenges. Phytochemicals delivery by nanocarriers also stand out as candidates to target and bio‐friendly therapy. Nanocellulose might fill in the gaps. Future research using nanomaterials targeting novel therapies/prophylaxis measures to COVID‐19 and future outbreaks is discussed.

## Graphical abstract

This systematic review addresses the potential and mechanisms of nanomaterials against the novel coronavirus. Nanocarbons, biopolymeric, copper, and silver nanoparticles stand out as potential antiviral and virucide agents toward self‐cleaning and reusable filter media/surfaces, drug administration, vaccines as prophylaxis/therapies options to Coronavirus disease 2019 (COVID‐19). Nanoflora can be an alternative with fewer side effects to treat COVID‐19.

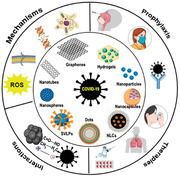

## 1 Introduction

A novel beta‐coronavirus, the novel coronavirus (SARS‐CoV‐2), or the severe acute respiratory syndrome coronavirus 2, was described as the causative agent of the pneumonia outbreak of coronavirus disease 2019 (COVID‐19), first reported in December 2019 in a local seafood market of Wuhan, Hubei province, China.^[^ ^1^, ^2^ ^]^ The same beta‐coronavirus genus of SARS‐CoV‐2 was earlier the causative of viral pneumonia pandemics caused by severe acute respiratory syndrome coronavirus (SARS‐CoV) and the Middle East respiratory syndrome coronavirus (MERS‐CoV) that emerged in 2002 in China and 2012 in the Arabian Peninsula, respectively.^[^ ^3^ ^]^ The World Health Organization reported, globally, as of 30 January 2021, more than 101 million confirmed cases of COVID‐19 and 2 196 944 deaths.^[^ ^4^ ^]^ Due to lockdown and quarantine, humanity is now facing its worst economic crisis since World War II,^[^ ^5^ ^]^ with a critical impact on healthcare and socioeconomic costs. Thus, there is a high demand to design tools to enhance antiviral strategies to control the spread of COVID‐19.

Viruses are considered a natural occurrence of nanoparticles due to their nanometric size. The genus beta‐coronavirus (as well as alpha‐, gamma‐, and delta‐coronavirus) is a member of the *Coronaviridae* family enveloped by spike (S) glycoproteins, positive‐sense, single‐stranded RNA, with virions of 118–140 nm essential for viral replication.^[^ ^6^ ^]^ The spike glycoprotein mediates the receptor‐binding spike protein and membrane fusion, responsible for viral cell entry in coronavirus infections.^[^ ^7^, ^8^ ^]^ Due to the small size and tunable surface charge, nanoscale materials have great potential to interact with spike protein to block viral infection initiation. Hence, it could be useful to design nanomedicine‐based strategies as novel antigens for nanovaccines, antiviral drugs, and immunomodulatory therapies for COVID‐19 management.^[^ ^9^ ^]^ If a nanomaterial can be modified with specific antiviral ligands (e.g., copper, zinc, silver (Ag)), these nanoantivirals could benefit COVID‐19 management.^[^ ^10^ ^]^ Besides, nanoparticle‐based antiviral drug delivery with high specificity can benefit the distinguishment of infected cells from healthy cells.^[^ ^11^ ^]^ An infection‐sensitive drug release based on nanoparticles could also minimize the premature drug release and loss drug before reaching the specific infection sites to overcome the challenge reported in the drug delivery design targeting intracellular infections.^[^ ^11^, ^12^, ^13^ ^]^

Researchers have shown an increased interest in nanomaterials for biomedicine, as antiviral drug delivery, due to their unique physical properties such as the small size (offering bioavailability and control of time release), tunable surface charge (for encapsulation of various drug types), and the large surface area to volume ratio (improving the solubility).^[^ ^14^ ^]^ Nanomaterials‐based platforms with nanoemulsions, carbon nanotubes, gold, and cobalt nanoparticles have been reported in several efforts against viruses outside and inside the host cells.^[^ ^15^, ^16^, ^17^ ^]^ For example, recent studies have demonstrated successful attempts with nanomaterials to control other viral human pathogens as human immunodeficiency virus type 1 (HIV‐1),^[^ ^18^, ^19^ ^]^ herpes simplex,^[^ ^16^ ^]^ hepatitis,^[^ ^20^ ^]^ influenza,^[^ ^21^ ^]^ and Zika virus.^[^ ^22^ ^]^ On the other hand, the increased novel variants and class of different viruses with antiviral drug resistance is now a challenge faced by the pharmaceuticals.^[^ ^23^, ^24^, ^25^ ^]^ Hence, broad‐spectrum antiviral compounds are needed. Thus, there has been a growing number of publications focusing on nanomaterials as antivirals by themselves. Silver nanoparticles were studied as antiviral agents for adenoviruses^[^ ^26^ ^]^ and media filter for airborne viruses.^[^ ^27^ ^]^ Silica and zinc oxide nanoparticles were reported as antiviral agents for agriculture.^[^ ^28^ ^]^ Furthermore, non‐toxic nanocellulose, extracted from biomass material cellulose, was reported as an ideal and green platform for drug delivery because of its safety, availability, and tunable surface chemistry.^[^ ^29^, ^30^ ^]^

Moreover, studies with phytochemicals derived from natural sources showed that bioactive compounds have already been effective against coronaviruses^[^ ^31^ ^]^ by increasing the immunity system or destroying the pathogens, which has received attention as an alternative source of drugs with fewer side effects.^[^ ^32^, ^33^, ^34^ ^]^ In this context, nanoflora—nanoparticles improving bioactive compounds′ bioavailability—was reported to enhance the delivery of insoluble phytochemicals repurposing antimicrobial drugs,^[^ ^35^, ^36^, ^37^ ^]^ including therapeutics approaches against viruses^[^ ^38^, ^39^, ^40^ ^]^ and and coronaviruses (CoVs).^[^ ^41^, ^42^ ^]^

**Figure 1:**
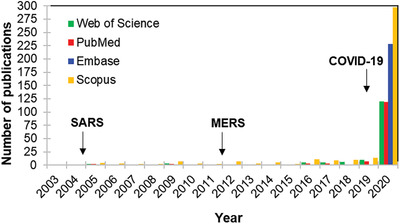
Publications indexed on Web of Science, PubMed, Embase, and Scopus. Keywords: “nano OR nanomaterials OR nanoparticle” and “coronavirus.” Search period: between January 1, 2004 and December 31, 2020.

However, only after the COVID‐19 emergence, a considerable number of papers has been published on nanomaterials‐based platforms and coronaviruses, although the first studies initiate after the first coronavirus pandemic, the 2002–2004 severe acute respiratory syndrome (SARS)—**Figure** **1** shows the number of publications retrieved in a simple search in some databases related to the topics “nano” and “coronavirus” between January 1, 2004 and December 31, 2020.

Since several studies are still undergoing, some overviews discussed nanomaterials′ potential as antiviral candidates for COVID‐19, but most took into account studies toward other CoVs.^[^ ^43^, ^44^, ^45^ ^]^ A meta‐analysis study has recently demonstrated nanoscale materials′ overall efficacy against other coronaviruses that appeared before the SARS‐CoV‐2 virus.^[^ ^45^ ^]^ The interactions between several viruses and graphene have prospected this nanostructure as a future possibility against COVID‐19^[^ ^46^, ^47^ ^]^ as disinfectants and antiviral coatings for personal protective equipment (PPE) for health workers.^[^ ^46^ ^]^ Once copper (Cu)‐based surfaces have also inactivated coronaviruses as human coronavirus 229E (HCoV‐229E)^[^ ^48^ ^]^ and SARS‐CoV‐2 in a short time compared to other surface materials,^[^ ^49^ ^]^ nanostructured containing copper, silver, and zinc have also been prospected to inactivate SARS‐CoV‐2 and manage COVID‐19.^[^ ^10^, ^50^, ^51^ ^]^ Moreover, due to its potent antiviral activity, the enrichment of plasma copper levels was recently hypothesized to boost innate and adaptive human immunity to prevent and treat COVID‐19.^[^ ^52^ ^]^

The primary aim of this systematic review (SR) is i) to identify, through a rigorous literature search analysis with a predefined strategy protocol, research using nanomaterials‐based approaches against the *Coronaviridae* family and the latest finds until December 31, 2020 toward the novel SARS‐CoV‐2 virus. Another objective of our study ii) is to critically analyze nanomaterials′ role as both a primary strategy (when the nanomaterials interact directly with the virus) and a secondary approach (when the nanomaterials improve the efficacy of another antiviral agent) against coronaviruses. Thus, this review also addresses the main finds in the anti‐coronavirus activity promoted by nanocarriers to identify potential candidates with minimum off‐target effects for COVID‐19 control. Some challenges and knowledge gaps on the design of antiviral nanoagents, in which the purposes are therapy, disinfectants, and antiviral surfaces (e.g., masks and coatings, for public uses), remain.^[^ ^53^, ^54^ ^]^ Nanomaterials′ modes of action to inhibit infection/inactivate SARS‐CoV‐2 have yet to be understood. Therefore, our study also contributes to understanding the interactions, types, and mechanisms of action associated between nanomaterials and SARS‐CoV‐2 inside and outside host cells.

## 2 Systematic Search Methods

This SR recovered and assessed all the data available in literature databases about nanomaterials′ antiviral potential to manage the COVID‐19 pandemic, from other coronaviruses to the novel SARS‐CoV‐2. To improve our SR quality, we followed a four‐phase flow diagram and the Preferred Reporting Items for Systematic Review and Meta‐Analyses (PRISMA) statement guidelines^[^ ^55^ ^]^ supported by the SR management StArt tool.^[^ ^56^ ^]^

### 2.1 Research Question

The focus question agrees to the problem, intervention, comparison, outcome, and study type (PICOS) strategy. As study type, we considered theoretical, hypothetical, computational, pre‐clinical, and clinical research. The research questions focused on as follows: Which nanoscale structures showed anti‐coronavirus effect potential for COVID‐19 control? Which nanocarrier has been reported to improve the use of antivirals agents against coronaviruses? What are the modes of action to inhibit viral infection and types of interactions between the viral surface and the nanosystem hypothesized/purposed to viral inactivation?

### 2.2 Search Sources and String Definition

Our search protocol strategy used search strings constructed and adapted for six electronic databases: Web of Sciences, PubMed, Embase, Scopus, SciFinder, and Science Direct. Besides, we performed additional searching on the reference list of relevant articles/reviews identified through the initial screening. The recovered papers of search sources were performed through a search string that summarizes the questions researched. The string was based on pre‐determined groups of keywords related to coronaviruses, nanoscale‐based structures, and their use as antiviral/virucide agents accordingly:

• *Search component 1*: nanoparticle* OR nanomaterial* OR nanostructure* OR CNT OR graphene OR graph* OR “silver nanoparticle” OR AgNp OR liposome OR “gold nanoparticles” OR silica OR “self‐assembly” OR nanocellulose OR hydrogel OR “nanoparticle‐based RNA” OR “copper nanoparticle”
• *Search component 2*: “SARS‐CoV‐2” OR CoV OR “nCoV‐2019” OR “COVID‐19” OR “enveloped viruses” OR virus* OR coronavirus OR Coronaviridae OR “SARS‐CoV” OR SARS
• *Search component 3*: antiviral OR virucide

### 2.3 Search Strategy, Selection Process, and Study Selection Criteria

The advanced search in the database was carried out considering research articles published in English between 2000 and 2020. The search started on July 14, 2020 and was updated from August 3 to December 31, 2020 to cover the maximum peer‐reviewed papers available, focusing directly on the novel SARS‐CoV‐2. The results of the screening were uploaded to the StArt tool. The authors first conducted the preliminary selection and extraction of data independently. **Table** **1** summarizes the inclusion/exclusion criteria adopted for the eligibility of studies. Further details can be seen in our previous work.^[^ ^57^ ^]^

**Table 1:** Inclusion/exclusion criteria on paper retrieving in this SR

Order	Step in PRISMA	Inclusion criteria	Exclusion criteria	On the basis of
1	Identification	Studies published in English	Reviews, letters to the editor, or editorials	Title, keywords and abstracts
2	Screening	Studies with anti‐coronavirus activity	No coronavirus was investigated.	
2		At least one nanostructured were studied	No nanoscale materials strategy was presented	
3		At least one segment of viral capture/inactivation was studied	Studies were genome sequencing	
4	Eligibility	–	Studies were preprints, not peer‐reviewed processed	
5		–	Studies did not match the purposes of our study	Full‐text reading

### 2.4 Data Extraction Process

For the data extraction of articles included in qualitative synthesis, the authors independently extracted and summarized the following information: nanomaterial type, size and shape, preparation strategy, coronavirus specie, significant results, interactions type between nanomaterial and coronavirus, mechanism of action, and potential application field.

### 2.5 Sources of Bias

We did not consider the draft/final form of papers deposited directly online at preprint servers that are not peer reviewed. Besides, the eligibility criteria and the impact of missing data might be considered sources of bias.

## 3 Main Findings

**Figure 2:**
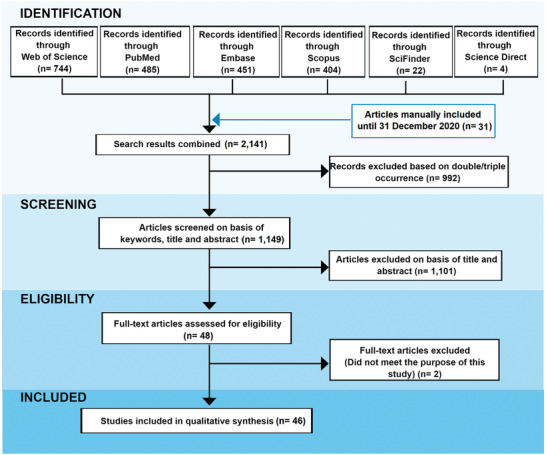
PRISMA flow diagram with results of the systematic search between 2000 and 2020.

The results of our systematic review were registered in a PRISMA flow diagram illustrated in **Figure** **2**. This systematic search identified a total of 744 papers at Web of Science, 485 at PubMed, 451 at Embase, 404 at Scopus, 22 at SciFinder, and 4 at Science Direct. Besides, we manually added a further 31 articles updating the search on the databases totaling 2141 papers. Of these, 992 were duplicates/triplicates and were excluded. A total of 1149 remained after the exclusion of repeated articles. After reading the titles, abstracts, and full‐text, only 46 papers were adequate for the current study purposes since they matched the eligibility criteria.

Of the 46 research papers retrieved for qualitative synthesis, several investigated nanomaterials potential against animal models and cell lines of 3 genera of *Coronaviridae* family causative illness in animals (*n* = 6) and humans (*n* = 6), including alpha‐coronavirus (*n* = 6), beta‐coronavirus (*n* = 5), and gamma‐coronavirus (*n* = 1). Related to 6 types of coronavirus originated in animals, were found 12 articles reporting nanomaterials against transmissible gastroenteritis virus (TGEV),^[^ ^58^ ^]^ avian infectious bronchitis virus (IBV),^[^ ^59^ ^]^ feline coronavirus (FCoV),^[^ ^60^ ^]^ murine hepatitis virus (MHV),^[^ ^61^ ^]^ type II feline infectious peritonitis virus (FIPV),^[^ ^62^, ^63^ ^]^ and porcine epidemic diarrhea virus (PEDV).^[^ ^64^, ^65^, ^66^, ^67^, ^68^, ^69^ ^]^ Concerning the 6 types of coronavirus cause illnesses in people were found 33 different studies reporting the role of nanomaterials against human coronavirus NL63 (HCoV NL63), human coronavirus OC43 (HCoV OC43),^[^ ^61^, ^70^ ^]^ human coronavirus 229E modified containing a renilla luciferase reporter gene (HCoV‐229E‐Luc),^[^ ^71^ ^]^ human MERS‐CoV,^[^ ^72^, ^73^ ^]^ human SARS‐CoV,^[^ ^74^ ^]^ and novel human SARS‐CoV‐2.^[^ ^52^, ^75^, ^76^, ^77^, ^78^, ^79^, ^80^, ^81^, ^82^, ^83^, ^84^, ^85^, ^86^, ^87^, ^88^, ^89^, ^90^, ^91^, ^92^, ^93^, ^94^, ^95^, ^96^, ^97^, ^98^, ^99^, ^100^ ^]^ One article was a meta‐analysis reporting the overall inhibition efficacy of nanomaterials against FIPV, F‐CoV, TGEV, H‐CoV, avian coronavirus (AvCoV), SARS‐CoV, and MERS‐CoV.^[^ ^45^ ^]^

We collected data from several nanostructures’ types studied against the coronavirus cited above as nanotubes, nanorods, nanoparticles, nanostars, nanowires, nanocrystals, nanosheets, nanogels, nanospheres, nanocapsules, nanoclusters, and nanostructured lipid carriers (NLCs). Of these, were identified the following nanotechnology‐based approaches: bioconjugated carboxyl quantum dots (QDs);^[^ ^74^ ^]^ silver nanomaterials;^[^ ^58^, ^60^, ^69^, ^80^, ^96^, ^97^ ^]^ synthetic virus‐like particles (sVLPs);^[^ ^77^ ^]^ gold nanoparticles;^[^ ^59^, ^77^ ^]^ biopolymeric/biodegradable polymeric nanoparticles and hydrogels (e.g., chitosan, collagen, poly(ethylene glycol) (PEG), poly lactic‐co‐glycolic acid (PLGA), and poly(hydroxyethyl) methacrylate);^[^ ^61^, ^62^, ^63^, ^79^, ^85^, ^86^, ^87^, ^91^, ^93^ ^]^ carbon quantum dots (CQDs)^[^ ^71^ ^]^ and cationic carbon dots based on curcumin (CCM‐CDs);^[^ ^68^ ^]^ glutathione(GSH)‐capped silver‐sulfide nanoclusters (GSH‐capped Ag_2_S NCs);^[^ ^64^ ^]^ gold nanorod‐based heptad repeat 1 (HR1) peptide;^[^ ^72^ ^]^ antigen and adjuvant‐loaded hollow polymeric nanoparticles;^[^ ^73^ ^]^ bovine serum albumin (BSA)‐coated tellurium nanoparticles (Te/BSA NPs) with a unique triangular star shape (Te/BSA nanostars);^[^ ^65^ ^]^ GSH‐modified zinc‐sulfide nanoparticles (ZnS NPs);^[^ ^66^ ^]^ glycyrrhizic‐acid‐based carbon dots (Gly‐CDs);^[^ ^67^ ^]^ multi‐walled carbon nanotubes (MWCNTs) with target functions;^[^ ^83^ ^]^ metal‐decorated single‐wall carbon nanotubes (SWCNTs);^[^ ^100^ ^]^ NLCs;^[^ ^88^ ^]^ graphene oxide;^[^ ^60^, ^82^, ^92^ ^]^ iron oxide nanoparticles (IONPs),^[^ ^75^ ^]^ functionalized graphene sheets;^[^ ^84^ ^]^ bimetallic nanorods (golden‐silver);^[^ ^69^ ^]^ titania nanoparticles (TNP);^[^ ^70^ ^]^ polyphosphate (Polyp) nanoparticle;^[^ ^91^ ^]^ nanosized formazans;^[^ ^78^ ^]^ copper nanoparticles/nanowires;^[^ ^52^, ^81^, ^92^, ^94^, ^95^ ^]^ silica nanoparticles (SiNPs);^[^ ^81^ ^]^ manganese nanodepot;^[^ ^89^ ^]^ decoy nanoparticles;^[^ ^90^ ^]^ besides nanocomposites between several of them.

**Figure 3:**
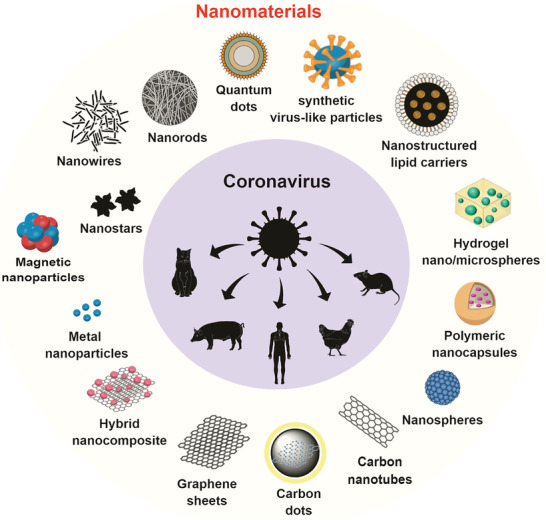
The main types of nanomaterials with antiviral properties studied against animal/human coronaviruses, including the novel SARS‐CoV‐2, found in the papers retrieved by this review.

In some papers, we identified the role of nanomaterials as a secondary approach against coronaviruses (*n* = 11), such as those which addressed active phytochemicals delivered by spherical nanoparticles as a natural, less toxic, antiviral agent against coronaviruses, which were categorized to be discussed in a separate section of this SR.^[^ ^61^, ^62^, ^63^, ^64^, ^66^, ^67^, ^68^, ^74^, ^80^, ^91^, ^93^ ^]^ We summarize in **Figure** **3** the main types of nanomaterials with anti‐coronavirus properties studies against animal and human coronaviruses found in papers retrieved by this SR.

This SR presented in the next section the review of our main finds with coronavirus and nanomaterial types, focusing on antiviral properties, as well as the chemical interface and cellular mechanisms associated with them.

## 4 Review of Literature Data Recovered

### 4.1 Nanomaterials against Causative Agents of Animal Coronaviruses

Several emerging viruses have been known to be transmitted from animals to humans. The scientific community argues that the most probable cause of the COVID‐19 pandemic started in the South China seafood market (Wuhan, Hubei) is zoonotic spillover.^[^ ^1^, ^2^ ^]^ Despite are still unclear which animals transmitted COVID‐19 to humans, recent findings show that SARS‐CoV‐2 has 96% genomic similarity with a bat coronavirus.^[^ ^101^ ^]^ Thus, these facts motivate us to choose articles and discuss the approaches with the anti‐coronavirus properties of nanomaterials of this section, given the possibility of extending the SARS‐CoV‐2 virus strategies to overcome the challenges of COVID‐19 control.

The type of nanomaterials found included nanoparticles, nanowires, nanostars, nanospheres, nanocapsules, nanoclusters (**Table** **2**). Most of them are inorganic, polymeric, and carbon‐based nanomaterials with varied morphology. These include spherical particles, rods, layers, triangular star shape, spherical particles capped with a corona‐like structure. The average size ranged from 5 to 140 nm.

**Table 2:** Nanomaterials approach with anti‐coronavirus activity against animal coronaviruses found in the retrieved papers

Nanomaterial	Average size	Shape	Strategy	Coronavirus	Application	Ref.
Ag^a)^ nanomaterials	Colloids: 10 nm Nanowires: 60–400 nm Nanoparticles: <20 nm	Spherical and wires	Silver (colloids, nanowires, and nanoparticles)	TEGV^b)^ (PUR46‐MAD)	Antiviral therapy	^[^ ^58^ ^]^
GO^c)^ GO‐Ag^d)^	GO^c)^: 0.6–9 nm GO‐Ag^d)^: 5–25 nm	GO^c)^: layers GO‐Ag^d)^: spherical	Silver anchored to graphene oxide sheets	FCoV^e)^ (NTU‐156)	PPE^f)^	^[^ ^60^ ^]^
Gold nanoparticles‐based sVLPs^g)^	100–140 nm	Like natural viral particles	Synthetic Av‐CoV‐IBV^h)^ spike protein antigen with 100 nm AuNPs^i)^	AvCoV‐IBV^h)^ (2575/98)	Vaccine immunology	^[^ ^59^ ^]^
Tellurium nanostars	57 nm	Triangular star shape	MES^j)^‐modified BSA^k)^‐coated tellurium nanostars	PEDV^l)^	Antiviral agents	^[^ ^65^ ^]^
Bimetallic nanorods	–	Rod like (core—shell)	Gold nanorods coated by Ag shell deposition	PEDV^l)^	Antiviral therapies	^[^ ^69^ ^]^

^a)^ Silver

^b)^ Transmissible gastroenteritis virus

^c)^ Graphene oxide

^d)^ Silver/graphene oxide nanocomposite

^e)^ Feline coronavirus

^f)^ Personal protection equipment

^g)^ Synthetic virus‐like particles

^h)^ Avian coronavirus infectious bronchitis virus

^i)^ Gold nanoparticles

^j)^ Mercaptoethane sulfonate

^k)^ Bovine serum albumin

^l)^ Porcine epidemic diarrhea virus.

**Figure 4:**
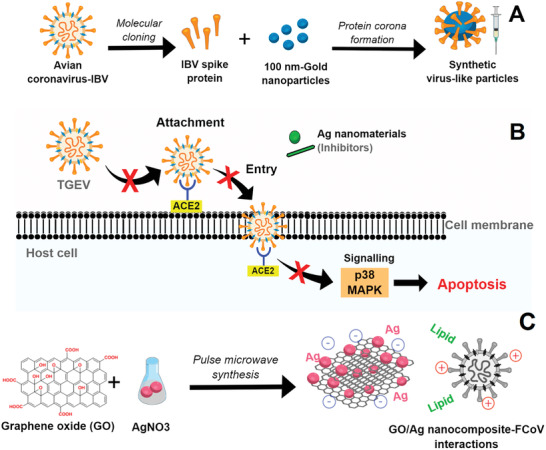
Main nano‐based approaches against animal coronaviruses found in this SR: A) sVLPs based on gold nanoparticles and incubation of IBV spike protein; B) inhibitory effects of silver nanomaterials on TGEV‐induced host cell infection and p38‐MAPK signaling activation; and C) interactions between Ag/GO nanocomposites and FCoV.

The high energy surfaces of synthetic nanoparticles were exploited to induce protein corona formation in sVLPs based on gold nanoparticles and incubation of IBV spike protein, obtained from the recombinant protein expression of IBV, as model antigens.^[^ ^59^ ^]^ The core–shell morphology binds with bulbous surface projections to mimetic natural viral particles and can be applied as a vaccine against AvCov‐IBV with an improved humoral and cell‐mediated immune response (**Figure** **4**A). Compared to the free antigen protein, the mechanism of the potent immunity response of sVLPs against AvCov‐IBV was attributed to primary lymphatic delivery and the multivalent antigen display, the major antibody titers, and the minor infection‐like symptoms.^[^ ^59^ ^]^

Although this approach presented a poor control of polydispersity (i.e., Ag nanowires varied from 20 to 400 nm), direct contact between Ag nanowires, Ag nanoparticles, and TGEV virus (a high mortality virus in seronegative suckling piglets) caused an inhibitory effect on viral infection and replication because of the interference with viral infection during attachment and entry (Figure 4B).^[^ ^58^ ^]^ The mechanism of action was studied and correlated with i) the inhibition of host cell apoptosis upregulation of p38/mitochondria‐caspase‐3 signaling route by Ag nanomaterials; ii) inhibition of the infection initiation through the direct interaction between Ag nanomaterials and the spike glycoprotein; and iii) Ag nanomaterials may alter the structure of surface proteins of TGEV/PEDV, inhibiting its identification and adhesion of the cellular receptor porcine aminopeptidase N.^[^ ^58^ ^]^ The insights provided by Ag nanomaterials into the antiviral therapy of coronaviruses inspired the development of a nanocomposite by anchoring spherical silver nanoparticle (AgNPs) with thin layers of GO sheets as an antiviral against the lipid enveloped FCoV, with 26% of viral inhibition at the minimum concentration, at a non‐cytotoxic level.^[^ ^60^ ^]^ Regarding the mechanism, in a general way, it was suggested that the unique structure of graphene oxide contribute as follow (Figure 4C): i) negatively charged GO might interact with positively charged lipid membranes inducing its rupture; ii) then, the lipid tail exposed could strongly bind with the aromatic plane of GO sheets; and finally iii) this GO sheets–lipid membrane interaction can attract more lipid membranes.^[^ ^60^, ^102^ ^]^ More recently, core–shell bimetallic nanorods were obtained by deposition of silver shell on gold nanorods (Au‐AgNRs) followed by releasing of Ag^+^ and exposure of Ag nanowires after endogenous reactive oxygen species (ROS) stimulation.^[^ ^69^ ^]^ The system inhibited PEDV replication by a multisite mechanism: i) inhibition of PEDV entry with decreasing mitochondrial membrane potential and caspase‐3 activity, and (ii) apoptosis induced by virus infection.^[^ ^69^ ^]^ Therefore, those attempts using silver nanomaterials provide insights into novel therapeutic strategies to prevent coronavirus replication.

Heparan sulfate (HS) proteoglycans are often used as a cellular attachment receptor to mediate viral infections′ adhesion and internalization. Inspired in this mechanism and in view to mimic the cell surface receptor HS, its analog mercaptoethane sulfonate (MES) was employed as the chemical modifier to synthesize Te/BSA nanostars with the advantage of upregulating its high antiviral activity against PEDV.^[^ ^65^ ^]^ This inorganic nanomaterial mechanism was correlated with ROS generation inhibition, highlighting its potential as a broad‐spectrum antiviral agent.^[^ ^65^ ^]^

Additionally, a meta‐analysis in a series of nanoscale materials in publications retrieved demonstrated a positive inhibition efficacy against animal coronaviruses in vitro and in vivo.^[^ ^78^ ^]^ We retrieved the other six original research articles with nanomaterials and natural compounds for treatment purposes against animal coronaviruses, which we decided to discuss later (see Section 4.4).

### 4.2 Nanomaterials against Causative Agents of Human Coronaviruses Identified before SARS‐CoV‐2

**Table** **3** shows the articles retrieved in this SR targeting the antiviral properties of CQDs, gold nanoparticles, titania oxide nanoparticles, and biopolymeric nanoparticles against HCoV‐NL63, HCoV‐OC43, SARS‐CoV, and MERS‐CoV.

**Table 3:** Nanomaterials approach with anti‐coronavirus activity as potential candidates against human coronaviruses identified before SARS‐CoV‐2 found in the retrieved papers

Nanomaterial	Average size	Shape	Strategy	Coronavirus	Potential application	Ref.
Chitosan nanospheres	10 nm–10 µm	Spherical	Genipin‐crosslinked chitosan	HCoV NL63^a)^, HCoV OC4^b)^	Adsorbents	^[^ ^61^ ^]^
CQDs^c)^ nanocrystals	4–9 nm	Spherical	Boronic acid‐functionalized CQDs	HCoV‐229E‐Luc^d)^	Antiviral drugs	^[^ ^71^ ^]^
Gold nanorods	18–54 nm	Rod	Gold nanorods‐based HR1^e)^ peptides	MERS‐CoV^f)^	Antiviral drugs	^[^ ^72^ ^]^
PLGA^g)^ hollow nanoparticles	114 nm	Spherical	Viral antigens and STING^h)^ agonists‐loaded hollow nanoparticles	MERS‐CoV^f)^ (EMC 2012)	Vaccine	^[^ ^73^ ^]^
TiO_2_ Nanoparticles	(Not reported)	Predominantly spherical	TNPs^i)^‐coated glass coverslips UVC radiation	HCoV NL63^a)^	Self‐cleaning surfaces	^[^ ^70^ ^]^

^a)^ Human coronavirus NL63

^b)^ Human coronavirus OC4

^c)^ Carbon quantum dots

^d)^ Modified human coronavirus 229E containing a renilla luciferase reporter gene

^e)^ HR1

^f)^ Middle East respiratory syndrome coronavirus

^g)^ Poly (lactic‐*co*‐glycolic acid)

^h)^ Stimulator of interferon genes protein

^i)^ Titanium dioxide nanoparticles.

Cationic chitosan nanospheres crosslinked by natural genipin (HTCC) presented high affinity with spike protein of coronaviruses and shown a more significant adsorbent effect against HCoV‐NL63 than HCoV‐OC43.^[^ ^61^ ^]^ This biopolymeric nanosphere was appointed as convenient candidates to remove coronaviruses from biological matrices and water with higher selectivity. Among other details that will be discussed later (see Section 4.4), the tremendous adsorptive capacity was attributed to HTCC‐virus electrostatic interactions and the high ionic strength caused by the HTCC cationization process.

Three types of CQDs were synthesized containing boronic acid groups, which proved to be essential, in a dose‐dependent manner, to the higher antiviral activity presented against the human coronaviruses HCoV‐229E‐Luc.^[^ ^71^ ^]^ These nanomaterials were suggested as potential candidates to replace the standard ribavirin/α‐interferon (α‐IFN), with fewer side effects. Concerning the mechanism of action, it was suggested that CQDs act by multistep at i) the initial stages of viral infection, inhibiting the viral entry, which probably is due to the inhibition of the interactions between the spike protein receptor and host cell membrane, caused by interactions between functional groups of CQDs with viral entry receptors; and ii) an equivalent extensive inhibition activity at the viral replication step.^[^ ^71^ ^]^

Aiming to develop an antiviral drug and vaccine against MERS‐CoV, gold nanorods^[^ ^72^ ^]^ and biopolymeric hollow nanoparticles made from poly (lactic‐*co*‐glycolic acid) (PLGA) with core–shell morphology^[^ ^73^ ^]^ were studied based on HR1 peptides and viromimetic STING agonists, respectively. In the first study, a series of HR1 peptides was developed by molecular docking to inhibit HR1/HR2‐mediated membrane fusion between the MERS‐CoV and host cells.^[^ ^72^ ^]^ Further, the nanoparticle‐based vaccine inhibited viral infection, was immunogenic and prevented the induction of undesirable lung disease in immunized human DPP4 enzyme transgenic mice.^[^ ^73^ ^]^ Recently, preliminary results with the virucidal effect of TNP against the HCoV‐NL63 and HCoV‐OC43 by photoactive TNP deposited on glass coverslips using UVC radiation. The authors also mentioned the potential to control the spread of COVID‐19 by self‐cleaning surfaces, and hence, they are extending the concept to SARS‐CoV‐2 in work currently underway.^[^ ^70^ ^]^

### 4.3 Nanomaterials and the Novel SARS‐CoV‐2: Recent Advances to Reduce the Spread of COVID‐19

**Figure 5:**
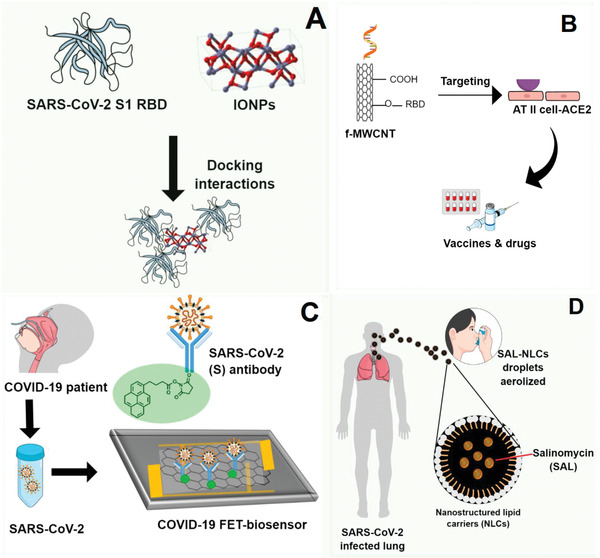
Nano‐based approaches against SARS‐CoV‐2 reviewed: A) docking interactions between FDA‐approved IONPs with spike RBD od SARS‐CoV‐2; B) functionalized CNTs with target functions; C) FET‐sensor with graphene sheets conjugated to SARS‐CoV‐2 spike antibody; D) pulmonary drug delivery system using NLCs to treat lung infected by SARS‐CoV‐2.

**Table** **4** displays computational approaches recently published repurposing medications, therapies, antiviral and immunologic agents to combat COVID‐19 patients. By molecular docking, the United States Food and Drug Administration (FDA)‐approved magnetic nanoparticles (Fe_2_O_3_ and Fe_3_O_4_) are repurposed to treat and control COVID‐19, based on its efficient hydrophobic interactions and hydrogen bonding with the chimeric S‐receptor‐binding domain (S1‐RBD) of SARS‐CoV‐2, to form a more stable complex^[^ ^75^ ^]^ (**Figure** **5**A). Likewise, novel gold nanoparticles functionalized with peptides formed a more stable complex with RBD than angiotensin‐converting enzyme 2 (ACE2).^[^ ^77^ ^]^ A non‐toxic approach with SiNPs showed that SiNPs‐encapsulated PolyP (to stabilize PolyP against alkaline phosphatase) inhibited the binding of ACE2 to S1‐RBD of SARS‐CoV‐2 at physiological concentration due to interactions between PolyP nanoparticles and amino acids on the surface of S1‐RBD.^[^ ^76^ ^]^ Moreover, this strategy was suggested to prevent and treat SARS‐CoV‐2 infection in the oropharyngeal and boost the immune system of thrombocytopenic COVID‐19 patients.^[^ ^76^ ^]^ Another study reported the potential of synthesized nano‐formazans as antiviral agents to manage COVID‐19 infection by docking simulations at a physiological solution: the results showed that formazan analogs could bind the active site 3CL protease of SARS‐CoV‐2, inhibiting the viral replication.^[^ ^78^ ^]^ Polymer nanoparticles‐optimized Remdesivir to repurpose it as antiviral therapy associated with lisinopril (a molecule of the therapeutic and lung‐protective effect of ACE) by Remdesivir‐loaded lisinopril‐functionalized PLGA.^[^ ^79^ ^]^ Thus, the potential of Remdesivir‐optimized nanoparticles was reported by a docking study, which confirmed interactions between lisinopril and ACE; and the binding of Remdesivir and RNA‐dependent RNA polymerase (RdRp), an enzyme involved in replication and transcription of the SARS‐CoV‐2 genome.^[^ ^79^ ^]^ Therefore, all these nanotechnologies approaches were predicted to interfere with viral adhesion to human host cell receptors and viral replication, thus inhibiting the viral infection.

**Table 4:** Computational approaches to predict ligand–receptor binding and structure‐based drug design for COVID‐19 management

Nanomaterial	Size	Strategy	Ligand–receptor binding results	Potential application	Ref.
Iron oxide nanoparticles	N/r^a)^	Nano‐mineral structure of Fe_2_O_3_ ^b)^ and Fe_3_O_4_ ^c)^	Interactions with S1‐RBD^d)^ of SARS‐CoV‐2^e)^	Repurposing medication	^[^ ^75^ ^]^
PolyP^f)^/Silica nanoparticles	210 ± 40 nm	Optimized polyP^f)^ encapsulated by SiNPs^g)^	Inhibition of binding of ACE2^h)^ to S‐protein SARS‐CoV‐2^e)^, at a physiological solution	Immunologic agents	^[^ ^76^ ^]^
Gold nanoparticles	N/r^a)^	Peptide‐functionalized gold nanoparticles	More stable complex with RBD^d)^ of SARS‐CoV‐2^e)^ than ACE2^h)^.	Antiviral agents	^[^ ^77^ ^]^
Nano‐sized formazans	23.75 ± 7.16 nm	Formazan analogs by dithizone and α‐haloketones reaction	Inhibition of SARS‐CoV‐2^e)^ chymotrypsin‐like protease, at a physiological solution	Antiviral agents	^[^ ^78^ ^]^
L‐PLGA NPs^i)^	N/r^a)^	Optimized Remdesivir‐loaded L‐PLGA NPs^i)^	Interactions Lisinopril‐ACE1^g)^ and remdesivir‐intracellular targeting protein RdRp^j)^	Antiviral therapy	^[^ ^79^ ^]^
Silver nanoparticles		Artemisinin, Artemether, and Artesunate delivery by silver nanoparticles	Interactions between negative charges of oxygen atoms of drugs with Ag surface	Antiviral drugs	^[^ ^80^ ^]^

^a)^ Not reported

^b)^ Iron(III) oxide or magnetite

^c)^ Iron(II,III) oxide or hematite)

^d)^ Chimeric spike‐receptor‐binding domain

^e)^ Novel coronavirus

^f)^ Ployp

^g)^ Silica nanoparticle

^h)^ Angiotensin‐converting enzyme inhibitor 1 or 2

^i)^ Lisinopril covalently grafted onto poly(lactic‐*co*‐glycolic acid) nanoparticles

^j)^ RNA‐dependent RNA polymerase.

**Table** **5** displays 19 recently published articles research focusing on novel coronavirus by theoretical, hypothesis, and applied research based on nanomaterials approaches to manage COVID‐19. Of these were found metal nanoparticles, carbon nanomaterials (e.g., carbon nanotubes, graphene layers, and graphene oxide), inorganic/organic polymeric nanoparticles, nanolipids, and nanodecoys purposing antiviral agents and surfaces, virucide agents, drug delivery, vaccines, immunological agents, and viral detection.

**Table 5:** Theoretical, hypothesis and applied research based on nanomaterials approaches to manage COVID‐19, found between 2000 and December 31, 2020

Nanomaterial	Shape, size	Strategy	Potential application	Ref.
SWCNTs^k)^	Cylinder	Metal‐decorated SWCNTs^k)^	PPE^l)^ design	^[^ ^100^ ^]^
GO^b)^	Layers	GO^b)^‐PMMA^m)^ film	PPE^l)^ design	^[^ ^82^ ^]^
MWCNTs^n)^	Multi‐walled cylinder	Acidizing and RNA Lyase‐modified carbon nanotubes	Vaccines and drugs	^[^ ^83^ ^]^
Copper	(Not reported)	Enrichment of plasma copper levels	Immunotherapy	^[^ ^52^ ^]^
Silica‐copper nanoparticles	Spherical	Silica‐copper/polymer (silicone or epoxy) nanocoating	Superhydrophobic self‐cleaning surfaces	^[^ ^81^ ^]^
CuNPs^a)^/GO^b)^ nanocomposite	Nanosheets, nanofibers	Electrospinning: multilayers of CuNPs^a)^/GO^b)^‐PLA^c)^ and CuNPs^a)^/GO^b)^‐CA^d)^ nanofibers	Respirator filter to antiviral face mask	^[^ ^92^ ^]^
Cu‐ZIF‐8^e)^/copolymer nanocapsule	Core–shell	Stabilizing CuNRs^f)^ by Pluronic F‐127^g)^, growth of ZIF‐8^e)^ to form a uniform core–shell structure	Filter media (reusable facial mask)	^[^ ^94^ ^]^
Shellac/CuNPs^a)^ nanohybrid	Spherical, ≈100 nm	Dual‐channel spray‐assisted nanocoating of shellac/CuNPs^a)^ to a nonwoven surgical mask	Photoactive antiviral mask (self‐cleaning)	^[^ ^95^ ^]^
AgNCs^h)^/silica nanocomposite	Predominantly spherical, <200 nm	AgNCs^h)^/silica‐based sputtered coating deposited on an FFP3 mask	Respirator facial masks, air filter	^[^ ^96^ ^]^
Silver nanoparticles	Spherical	Colloidal coating: silver nanoparticles dispersed in PVP^i)^	Virucide coating	^[^ ^97^ ^]^
Aluminum nanofibers	Fibers	Nanostructured aluminum alloy surface by wet etching	Antiviral surfaces	^[^ ^98^ ^]^
Gold nanoparticles	Spherical, 2.4 nm	Sulfonated gold nanomaterials: coating with MUS^j)^ ligands	Antiviral agents	^[^ ^99^ ^]^
Graphene sheets	Layers	PBASE^o)^‐modified graphene sheets FET^p)^ sensor with graphene sheets conjugated to SARS‐CoV‐2^q)^ spike antibody	Immunodetection	^[^ ^84^ ^]^
Polymeric nanoparticles	Spherical	Bioinspired DNase‐I^r)^‐coated melanin‐like nanospheres: recombinant DNase‐I^r)^/PEG^s)^ coating	Therapy for with ARDS^t)^ or sepsis in severe COVID‐19 patients	^[^ ^85^ ^]^
Polymeric nanoparticles	Spherical, 220 nm	DNase‐I^r)^‐coated polydopamine‐PEG^s)^ nanoparticles (exogenous administration)	Therapy for with ARDS^t)^ or sepsis in severe COVID‐19 patients	^[^ ^86^ ^]^
Polymeric nanoparticles	(Not reported)	Ivermectin‐delivery by (PLGA‐*b*‐PEG‐Mal)^u)^ copolymer nanoparticles (orally administrable)	Antiviral drug	^[^ ^87^ ^]^
Nanostructured lipid carriers	Spherical	Pulmonary delivery of Salinomycin by nanostructured lipid carriers	Drug delivery	^[^ ^88^ ^]^
Manganese nanodepot	(Not reported)	Droplet‐confined nanoprecipitation in water‐in‐oil micro‐emulsion + thin‐film dispersion method	Vaccine adjuvant	^[^ ^89^ ^]^
Decoy nanoparticles	(Not reported)	Fusing genetically engineered cell membrane nanovesicles (293T/ACE2 and THP‐1cells)	Therapeutic vaccines	^[^ ^90^ ^]^

^a)^ Copper nanoparticles

^b)^ Graphene oxide

^c)^ Polylactide (as matrix)

^d)^ Cellulose acetate (as matrix)

^e)^ Copper^2+^‐doped zeolitic imidazolate framework‐8

^f)^ Copper nanowires

^g)^ Block copolymer of PEG‐PPG‐PEG structure (PEG: poly(ethylene glycol); PPG: poly(propylene glycol))

^h)^ Silver nanoclusters

^i)^ Polyvinylpyrrolidone

^j)^ Decanesulfonic acid

^k)^ Single‐walled carbon nanotubes

^l)^ Personal protection equipment

^m)^ Poly(methyl methacrylate (as matrix)

^n)^ Multi‐walled carbon nanotubes

^o)^ 1‐Pyrenebutyric acid *N*‐hydroxysuccinimide ester

^p)^ Field‐effect transistor

^q)^ Novel coronavirus

^r)^ Deoxyribonuclease I

^s)^ Poly‐(ethylene glycol)

^t)^ Acute respiratory distress syndrome

^u)^ Poly(lactide‐*co*‐glycolide)‐block‐poly‐(ethylene glycol)‐maleimide nanoparticles.

**Table 6:** Recent hypotheses, computational studies, and pre‐clinical/clinical research into bioactive compounds and nanomaterials against coronaviruses

Nanomaterial	Size	Shape	Phytochemical	Coronavirus	Potential application	Ref.
Carboxyl quantum dots	20 nm	Spherical	Catechin gallates	SARS‐CoV^a)^	Antiviral agent	^[^ ^74^ ^]^
Chitosan‐gel nanospheres	60 nm	Spherical	Genipin	HCoV‐NL63^b)^ HCoV‐OC43^c)^ MHV^d)^	Adsorbents	^[^ ^61^ ^]^
PEG‐PLGA^e)^ nanocapsules	36 nm	Spherical	Diphyllin	FIPV^f)^	Antiviral drugs, immunotherapy	^[^ ^62^ ^]^
CCM‐CDs^g)^	1.5 nm	Spherical	Curcumin	PEDV^h)^	Antiviral drugs	^[^ ^68^ ^]^
Chitosan nanoparticles	250–450 nm	Spherical/Cuboidal	Curcumin	FIPV^f)^	Antiviral drugs	^[^ ^63^ ^]^
CDs^i)^	11.4 nm	Spherical	Glycyrrhizin	PEDV^h)^	Antiviral drugs	^[^ ^67^ ^]^
Ag_2_S‐NCs^j)^	5.3 nm	Spherical	Glutathione	PEDV^h)^	Antiviral drugs	^[^ ^64^ ^]^
ZnS‐NPs^k)^	3.8 nm	Spherical	Glutathione	PEDV^h)^	Antiviral drugs	^[^ ^66^ ^]^
Collagen hydrogel	50–100 nm	Fibrils	Polyp nanoparticles	SARS‐CoV‐2	Antiviral agent, Immune supplementation	^[^ ^91^ ^]^
Polymeric hydrogel	–	–	Griffithsin	SARS‐CoV‐2	Ophthalmic drug delivery	^[^ ^93^ ^]^
Silver nanoparticles	–	–	Artemisinin, Artemether, Artesunate	SARS‐CoV‐2	Antiviral drugs	^[^ ^80^ ^]^

^a)^ Severe acute respiratory syndrome coronavirus

^b)^ Human coronavirus NL63

^c)^ Human coronavirus OC43

^d)^ Murine coronavirus

^e)^ Poly(ethylene glycol‐block‐poly‐(lactide‐coglycolide)

^f)^ Type II feline infectious peritonitis virus

^g)^ Cationic carbon dots based on curcumin

^h)^ Porcine epidemic diarrhea virus

^i)^ Carbon dots

^j)^ Silver‐sulfide nanoclusters

^k)^ Zinc‐sulfide nanoparticles). Polyhydroxyethylmethacrylate.

Carbon nanotubes were present in an innovative theoretical proposal to develop vaccines and drugs for COVID‐19, exploiting coronavirus physical–chemical properties and constructing a target acidification environment (Figure 5B). MWCNTs with target functions, RNA lyase‐ and acid‐functionalized combined with photothermal effects, were hypothesized to block viral infection and replication routes in the host cells.^[^ ^83^ ^]^ Regarding modes of actions, acidizing the cell environment, generating a photodynamic thermal effect by irradiation of functionalized‐carbon nanotubes, and smart drug‐delivery of a viral RNA lyase destruction were proposed.^[^ ^83^ ^]^ Free‐energy calculations by DFT were performed on carbon‐based nanomaterials and were proposed to develop nanodevices useful in COVID‐19 and future pandemics management.^[^ ^82^, ^100^ ^]^ Exploiting ROS molecules′ harmfulness to coronavirus, nanofilters based on metal‐decorated (Pt, Cu, Rh, and Ru)‐SWCNTs, combined with H_2_O_2_, were presented as an effective platform for future experiments in SARS‐CoV‐2 absorption.^[^ ^100^ ^]^ The nanocomposite use for elastomeric respirators and self‐cleaning surfaces could be used in hospitals during future pandemics.^[^ ^100^ ^]^ Further experiments combined electrostatic composite films (coagulated GO‐PMMA) with water aerosols to create a nanocomposite surface that generated a negative voltage from water evaporation. Hence, an electrostatic bond with coronaviruses′ spike protein.^[^ ^82^ ^]^ In the absence of the novel coronavirus to test, the study used the model beer yeast cells. The mechanism appears to be linked to electrostatic interactions between the nanosystems (due to their nanometric size and chemical character) with the coronaviruses′ negatively charged spike proteins. DFT calculations showed a generous capacity of metal‐decorated SWCNTs for peroxide and hydroxyl radical capture with a very long recovery time.^[^ ^100^ ^]^ Once in contact, the functional groups of GO layers and water molecules interact, thus generating an extra electric field induced through the heterostructure formation with an enhanced dipolar redistribution at the interface.^[^ ^82^ ^]^

The potential of Cu to neutralize infectious viruses and induce viral killing mediated by ROS^[^ ^52^ ^]^ have been hypothesized to booster immune system against COVID‐19 by Cu supplementation^[^ ^52^ ^]^ and motivated researchers to formulate copper to fabricate nanodevices as antiviral, self‐cleaning, and reusable filter media/surfaces to prevent the COVID‐19 spread.^[^ ^81^, ^92^, ^94^, ^95^ ^]^ A superhydrophobic coating based on silica/copper nanoparticles dispersed in silicone or epoxy polymer (flexible, superhydrophobic, and regenerative monolith surfaces) was hypothesized to the self‐cleaning surface for implementation in public and healthcare work environments to eradicate SARS‐CoV‐2 spread and protect against COVID‐19 by three‐step strategy: i) virus encapsulation, ii) contamination suppression, and iii) virus elimination.^[^ ^81^ ^]^ Although face masks can limit transmission, the increased demand for disposable masks also demand many resources and has raised concerns about the generation of waste.^[^ ^94^ ^]^ Thus, some strategies using sustainable polymers have been studied to reduce transmission and the impact of waste. A filtration system from a nanofibrous respirator facial mask containing multilayers of Cu nanoparticles/GO nanosheets was dispersed in a nanofibrous matrix of biodegradable polylactic acid (PLA) or cellulose acetate (CA).^[^ ^92^ ^]^ Interestingly, the use of thermoplastic polymers such as PLA and CA can provide a stable fit with face anatomy.^[^ ^92^ ^]^ Likewise, low‐cost scalable synthesis of Cu nanowires/ZIF‐8 stabilized by an amphiphilic block copolymer (Pluronic F‐127) in a core–shell structure was direct deposited onto a reusable face mask system and produced 55% inhibition of SARS‐CoV‐2 replication after 48 h at a concentration of 1 µg.^[^ ^94^ ^]^ A dual‐channel spray‐assisted nanocoating hybrid of shellac/CuNP to a photoactivated antiviral facial mask with self‐sterilizing and reusability was reported with virucide effects.^[^ ^95^ ^]^ Another nanotechnology‐based filter air was reported with silver nanoclusters/silica composite sputtered coating applied on FFP3 mask with virucide effect, and completely reduced SARS‐CoV‐2 titer to zero on tested conditions to the sample with the highest content of Ag. Other research that addressed the antiviral and virucide effect of silver discussed the interaction between coating‐based colloidal AgNPs and SARS‐CoV‐2 by Luciferase‐based pseudovirus entry assay and revealed that Ag nanomaterials potently block viral entry step via disrupting viral integrity.^[^ ^97^ ^]^ Furthermore, SARS‐CoV‐2 was wholly inactivated after 6 h of exposure to a nanostructured aluminum alloy surface obtained by wet‐etching technique.^[^ ^98^ ^]^

Gold nanoparticles coated by decanesulfonic acid ligands inhibited the activity of authentic SARS‐CoV‐2 in a nanomolar range, and contrarily to most of the strategies that targeted the inhibition of SARS‐CoV‐2 cell entry by blocking spike protein‐ACE2 receptor binding, this sulfonated nanomaterial can inhibit SARS‐CoV‐2 attachment by blocking spike protein‐HS receptors binding and was suggested as simply reversible and potent antiviral agents.^[^ ^99^ ^]^

A simple, highly selective, sensitive, and rapid method for detecting the SARS‐CoV‐2 virus in nasopharyngeal swab samples from COVID‐19 patients, without sample pre‐treatment/labeling, was performed on a field‐effect transistor (FET)‐based biosensor using functionalized‐graphene sheets as a receptor^[^ ^84^ ^]^ (Figure 5C). The sensor target detected the SARS‐CoV‐2 antigen protein, cultured SARS‐CoV‐2 virus, and SARS‐CoV‐2 from clinical samples.^[^ ^84^ ^]^ The authors proposed a high dependence between SARS‐CoV‐2 spike protein and specific binding with the SARS‐CoV‐2 antigen, and the chemically modified graphene surface promoted this binding affinity through a pyrene backbone with an electron‐withdrawing group.^[^ ^84^ ^]^

Due to the biocompatibility nature and size of NLCs, a study hypothesized the pulmonary delivery of Salinomycin (SAL) carried by NLCs as a promising candidate to treat COVID‐19 patients^[^ ^88^ ^]^ (Figure 5D). The SAL encapsulation by NLCs sounds like a potential strategy to increasing its absorption at the local infection due to the good aerodynamical properties of NLCs, which could be aerosolized by droplets as antiviral drugs. Besides, the hypothesis was based on pieces of evidence that SAL has the potential to prevent the viral entry into the cytosol, prevent membrane fusion in a pH‐dependent way, interact with spike protein, inducing the ACE2 binding, and preventing the release of viral acid nucleic into the cytoplasm.^[^ ^88^, ^103^, ^104^ ^]^ Ivermectin is a clinically approved antiviral drug and was repurposed against SARS‐CoV‐2, using orally administering PLGA‐grafted‐PEG‐maleimide nanoparticles, an amphiphilic and biodegradable block copolymer system, which was capable of delivering a more potent therapeutic dose.^[^ ^87^ ^]^ The system demonstrated potential for the therapeutic drug to COVID‐19 by multisite inhibition into decreasing the viral uptake and transmission by i) inhibition of viral spike protein level and its entry rate by downregulation of ACE2 expression, and ii) possibly, inhibition of nuclear transport activities mediated by proteins (e.g., importin α/β1 heterodimer).^[^ ^87^ ^]^

A recent study hypothesized that excessive neutrophil extracellular traps (NETs) and extracellular DNAs (eDNAs) could activate NETosis, neutrophil‐specific programmed cell death might be associated with COVID‐19 pathogenesis.^[^ ^85^, ^105^ ^]^ Nowadays, there are no FDA‐approved antiviral medications that can effectively suppress the SARS‐CoV‐2‐mediated neutrophil activities, cytokine storm, acute respiratory distress syndrome (ARDS), and sepsis, thus, promoting widespread patient improvement.^[^ ^85^, ^86^ ^]^ Therefore, different strategies using polymeric nanoparticles to deliver antiviral agents have been evaluated to drug repurpose.^[^ ^85^, ^86^, ^87^ ^]^ An in vivo study with a septic mouse model showed the potential of bioinspired DNase‐I‐coated melanin‐like nanospheres using PEG to reduce neutrophil counts and modulate sepsis‐associated NETosis dysregulation in the plasma of COVID‐19 patients alleviating inflammation and mortality.^[^ ^85^ ^]^ Further research showed an exogenous administration of a long‐acting DNase‐1, a recombinant DNase‐1‐coated polydopamine‐PEG nanoparticulated, can reduce SARS‐CoV‐2‐mediated neutrophil activities and cytokine storm as a potential treatment to COVID‐19‐related illnesses.^[^ ^86^ ^]^

Manganese nanodepot (nanoMn) and decoy nanoparticles were proposed as simple, safe, and robust vaccine adjuvants and antiviral agents to manage COVID‐19.^[^ ^89^, ^90^ ^]^ Although manganese can reduce IFN response—a central host response against viruses—there is a challenge in its applicability due to non‐specific distribution and neurotoxicity.^[^ ^89^ ^]^ Thus, manganese was repurposed in nanoMn with enhanced cell uptake and persisted release of Mn^2+^ in a pH‐sensitive manner, boosted IFN response, broad‐spectrum in vitro and in vivo antiviral effects and macrophage polarization; no neuroinflammation effects were observed; nanoMn acted as a vaccine adjuvant to boost host adaptive immunity.^[^ ^89^ ^]^ Otherwise, a decoy nanoparticle made by genetic engineering can protect host cells against COVID‐19 infection by a two‐step neutralization approach: i) first, virus neutralization followed by ii) inflammatory cytokine neutralization in the second step (e.g., interleukin 6 and granulocyte‐macrophage colony‐stimulating factor).^[^ ^90^ ^]^ Thus, the authors reported stabilization of ACE2 expression; protection of host cell against infection by competition between nanodecoy and host cell to SARS‐CoV‐2 binding; and suppressing immune disorder and lung injury in an acute pneumonia mouse model by nanodecoy through in vivo assay.^[^ ^90^ ^]^

### 4.4 Natural Bioactive Compounds and Nanoparticles against Coronavirus and SARS‐CoV‐2: A Promissory, Healthy, and Bio‐Friendly Strategy for Drug Delivery

Eleven of the 45 articles retrieved highlighted strategies based on nanomaterials as a secondary approach to enhance natural compounds′ use, improving their bioavailability, solubility, and antiviral activity. Phenolic compounds, glycosides, terpenes, saponins, peptides, and proteins can play an essential role against viruses from the *Coronaviridae* family. Of these, we identified genipin, diphyllin, curcumin, glutathione, glycyrrhizic acids, polyP nanoparticles, and griffithsin.^[^ ^61^, ^62^, ^64^, ^66^, ^67^, ^68^ ^]^

The expert opinion reported that morphology characteristics (size and shape) are a fundamental aspect of nanoparticle design, once it is directly connected with pharmacokinetics and cell uptake for drug delivery purposes, especially for those with minimal side effects.^[^ ^106^ ^]^ In the case of drug carriers in blood for lymphatics channels, the geometry and aspect ratio of shape plays a crucial role in how nanoparticles will be transported. From **Table** **6**, we note that most of all nanoparticles studied are spherical and presented an impressive average size varying from 11.4 to 1.5 nm in the last 3 years reported attempts.^[^ ^63^, ^64^, ^66^, ^67^, ^68^, ^91^, ^93^ ^]^

**Figure 6:**
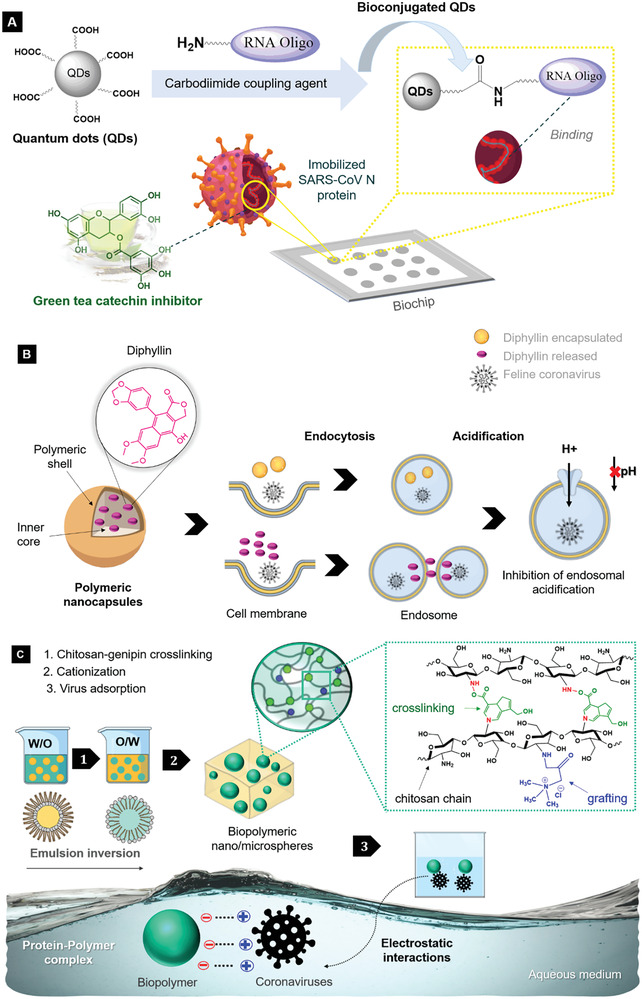
Schematic of preparation and mechanisms of phytochemicals/nanoparticles against coronaviruses reviewed: A) antiviral activity of catechins on a bioconjugated QDs‐based biochip by interactions with SARS‐CoV N protein; B) diphyllin encapsulated by polymeric nanoparticles as a potential therapeutic target against FCoV (V‐ATPase inhibition); C) viral adsorption on the surface of biopolymeric nano/microspheres by electrostatic interactions with HCoV‐NL63 and MHV S protein.

**Figure** **6** shows a schematic representation of some strategies reviewed on active phytochemicals (a broad‐spectrum “host‐targeted” antiviral)/nanoparticles systems as promising candidates against coronaviruses as virus adsorbents, antiviral agents. and immunomodulatory drugs. The amide coupling reaction is often used in medicinal chemistry^[^ ^107^ ^]^ to generate novel compounds for antiviral drug discovery.^[^ ^19^, ^108^ ^]^ Carboxyl quantum dots, QD650, has carboxylic acid terminal groups that could efficiently covalently conjugate with amine groups of biomolecules (e.g., proteins, nucleic acids), forming an amide bond through carbodiimide‐mediated coupling reactions. Herein, we reviewed a nanostructure approach against the beta‐coronavirus SARS‐CoV to rapidly identify natural inhibitors screening of the SARS‐CoV nucleocapsid (N) protein, beyond an optical method based on carboxyl quantum dots‐conjugated RNA nucleotides system, with applicability for imaging analysis on a biochip^[^ ^74^ ^]^ (Figure 6A). The systems were used as a platform for natural inhibitors based on several polyphenols, in which (‐)‐catechin gallate and (‐)‐gallocatechin gallate, obtained from green tea (*Camellia sinensis*), presented the higher inhibition effect against SARS‐CoV N protein (more than 40% inhibition at 0.05 µg L^−1^).^[^ ^74^ ^]^ The vacuolar‐ATPase (v‐ATPase) dysregulation has already been discussed and linked with drug resistance in viral infections^[^ ^109^ ^]^ and cancer therapies.^[^ ^110^ ^]^ Hence, v‐ATPase blocking could be an attractive target for antiviral approaches. A nanoformulation with diphyllin (a lignan obtained from the plant *Cleistanthus collinus)* encapsulated by biocompatible block copolymer nanocapsules of PEG‐PLGA exhibited a potent inhibitory effect against FCoV in fcwf‐4 cells^[^ ^62^ ^]^ (Figure 6B). The strategy was studied using a nanoparticulated system with diphyllin as a novel v‐ATPase blocker as an alternative for the bafilomycin A1, given its compromised clinical applicability due to its high cytotoxicity, low water solubility, and potent off‐target effect.^[^ ^109^ ^]^ Besides that, genipin from *Gardenia jasminoides* was found as a crosslinker agent of chitosan nano/microspheres (HTCC), that was further cationized and studied as adsorbents (in aqueous suspension medium) with high selectivity against humans and animal coronaviruses (HCoV‐NL63 and MHV)^[^ ^61^ ^]^ (Figure 6C). An advantage of this attractive water‐soluble nanoparticle with genipin and chitosan as an antiviral strategy is that booth are non‐toxic materials.

Curcumin pyrolysis applied to prepare uniform and stable CDs with wealthy hydrophilic groups,^[^ ^68^ ^]^ a glycyrrhizic acid‐based carbon dots,^[^ ^67^ ^]^ glutathione‐capped Ag_2_S nanoclusters,^[^ ^64^ ^]^ and glutathione‐modified zinc‐sulfide nanoparticles,^[^ ^66^ ^]^ were developed with high effectiveness against PEDV coronavirus. Curcumin is a polyphenol obtained from *Curcuma longa* that can play a vital role in antiviral activity due to its phenolic hydroxyl groups. Once the low bioavailability impairs its therapeutic application, curcumin was encapsulated in chitosan nanoparticles by ionic gelation technique with improved in vitro antiviral activity and bioavailability by oral administration in cats infected with FIPV.^[^ ^63^ ^]^

PolyP, a non‐toxic and non‐immunogenic inorganic polymer derived from marine bacteria, was added into a mucin/collagen‐based hydrogel to simulate the mucus of the nasopharynx and bronchial epithelium on human alveolar basal epithelial A549 cells.^[^ ^91^ ^]^ This strategy was suggested to stimulate an innate antiviral response by improved mucin barrier (high in antimicrobial proteins) and as a potential antiviral agent by exerting protection against SARS‐CoV‐2‐cell attachment.^[^ ^91^ ^]^ Griffithsin is a small lectin derived from red algae (*Griffithsia* spp.) of broad‐spectrum antiviral activity against coronavirus.^[^ ^111^, ^112^ ^]^ Thus, poly (2‐hydroxyethyl methacrylate) hydrogel lenses with nanoparticles releasing griffithsin were hypothesized as therapeutic contact lenses to protect healthcare workers′ ocular surface as extra protection in daily practice against COVID‐19 infection.^[^ ^93^ ^]^ The synergistic potential between antimalarial drugs as Artemisinin, Artemether, and Artesunate—natural sesquiterpene lactones from wormwood plant (*Artemisia annua*)—coated with antiviral AgNPs was optimized in a structure by molecular dynamics and suggested to improve the permeability and time retention of these drugs enhancing the therapeutic action against malaria and COVID‐19.^[^ ^80^ ^]^

Related to the mechanism of action, the green tea polyphenols, as epicatechin gallates, were reported as a potent antiviral entry inhibitor capable of blocking the host's binding of glycoprotein CD4 cell with glycoprotein gp120 of HIV‐1 and hence, preventing the viral infection.^[^ ^113^ ^]^ The antiviral activity of green tea catechins was suggested in previous studies against other enveloped viruses. It was mainly attributed to its hydroxyl, galloyl, and pyrogallol groups,^[^ ^114^ ^]^ as well as phenolic OH groups on B‐ring,^[^ ^115^ ^]^ which can act at several stages of the viral entry:^[^ ^113^ ^]^ i) affecting the expression of viral antigens or ii) inhibiting the genome replication. The desorption properties of HTCC was explained in terms of i) electrostatic Coloumb attraction of the genipin–chitosan derivatives nanospheres with the spike protein of HCoV‐NL63, which can form a protein–polymer complex, resulting in virus neutralization; and ii) the high ionic strength promoted by chitosan cationization.^[^ ^61^ ^]^ In a dose‐dependent way, the diphyllin inhibited endosomal acidification affecting the viral cellular susceptibility and inhibits the downstream coronavirus replication.^[^ ^62^ ^]^ Furthermore, multisite inhibition mechanisms were found for all approaches with GSH, curcumin, and glycyrrhizin tested against PEDV: i) inhibition of viral entry by changing the structure of the viral surface protein, prevent the viral RNA synthesis and budding; ii) suppression of the ROS generation; and iii) suppression of viral reproduction by activation of IFN‐stimulating genes and the expression of pro‐inflammatory cytokines. Likewise, it was hypothesized that the griffithsin's capacity to block viral entry^[^ ^93^ ^]^ due to its high affinity to glycoproteins sites of MERS‐CoV^[^ ^111^ ^]^ and SARS‐CoV.^[^ ^112^ ^]^ PolyP has already been suggested to block SARS‐CoV‐2‐cell attachment by blocking the binding of RBD of spike protein of SARS‐CoV‐2 to ACE2 cell receptor in vitro.^[^ ^76^, ^91^ ^]^ Density functional theory (DFT) calculations predicted the highest affinity of antimalarial drugs to interact with AgNPs surfaces in the order Artesunate > Artemisinin > Artemether, due to the more negative charges on O_6_ atom of Artesunate, O_5_ atom of Artemisinin, and O_3_ atom of Artemether.^[^ ^80^ ^]^

## 5 Conclusions and Outlook

In this systematic review, the papers retrieved and analyzed show silver, copper, and polymer‐based nanomaterials as the primary with efficient anti‐SARS‐CoV‐2 properties. The strong virucide potential of copper and silver nanomaterials with varied morphology and fabrication form were prospected to make reusable and self‐cleaning surfaces (e.g., respirator facial masks and coating surfaces) in healthcare work environments to reduce the spread of COVID‐19. Biopolymeric and biodegradable polymers in nanoformulations have prospected drug delivery against SARS‐CoV‐2, as well as nanodecoy and manganese nanoparticle was suggested as a simple, safe, and robust technology for vaccine adjuvants or antiviral agents once it increased immune response by in vivo assays. Virucide surfaces and adsorbents to capture/inactivate SARS‐CoV‐2 and other beta‐CoV were also proposed as adsorbents for coronavirus inactivation. Cutting‐edge nano biosensors technologies, nanostructured carriers for pulmonary drug delivery, sVLPs, and polymeric hydrogels were pointed out as potential agents for antiviral drugs, therapeutic vaccines, and immuno‐based therapies. A study with real clinic samples of COVID‐19 patients demonstrated graphene use for immunodiagnostic assay with high specificity and celerity, potentially useful for serological tests and detection of infection.

Molecular docking and dynamic simulations are powerful tools to study the relationship between receptor–ligand binding affinity in drug discovery using nanomaterials. Therefore, all the nanotechnologies studied by computational tools were predicted to interfering with SARS‐CoV‐2 adhesion to human host cell receptors and viral replication, thus inhibiting the viral infection.

Primary and secondary metabolites of plants and microorganisms (e.g., phenolic, terpenes, glycosides, polysaccharides, and Polyp), well‐known antimicrobial compounds, delivered by spherical nanoparticles, were a bio‐friendly and potential strategy to produce antivirals therapies for coronavirus once nanomaterials enhanced their solubility, bioavailability, and antiviral activities. The phenolic phytochemicals acted as multisite inhibitors at several stages of the viral entry, affecting viral antigens′ expression, or inhibiting the genome replication. Biodegradable polymeric nanoparticles are non‐toxic and biocompatible options for drug delivery. Due to its high surface energy, the immense possibility of functionalization, and their strong‐binding amino acids character, silver and carbon‐based nanomaterials showed high potential to be used in different segments to control the spread of COVID‐19. Thus, it showed booth a fundamental role and a secondary action (as carriers and antivirals).

Understanding the interface between nanomaterials and coronaviruses reviewed is fundamental to designing target antivirals for COVID‐19 infection. Their varied morphology, chemical diversity, excellent physical–chemical properties, and the possibility of binding several types of compounds in their surface with target functions, as well as the synergism between them (e.g., nanocapsules and nanocomposites), justified nanomaterials′ as potential nanomedicine and prophylactics tools against COVID‐19. Nanocarbons can act against coronaviruses multivalent interactions (e.g., electrostatic interactions, hydrogen bonds, and hydrophobic interactions) with spike protein and lipid tails, destroying the membrane, blocking cell entry and viral replication. The primary mechanism to block cell viral infection inhibited SARS‐CoV‐2 cell entry by blocking the binding between RBD of spike protein and the human ACE2 receptor. However, sulfonate ligands on the gold nanoparticle surface can inhibit the SARS‐CoV‐2 cell attachment by inhibiting the binding between spike protein and HS receptors. Many strategies can suppress ROS generation, inhibit host cell apoptosis, inhibit endosomal acidification, acting as multistep inhibitors on viral entry and replication. Photoactivated copper, silver, and TNP showed the highest potential as virucidal agents.

No study using the eco‐friendly nanocellulose was retrieved. That was surprising, considering that nanocellulose is sustainable, non‐toxic, antimicrobial, biocompatible, relatively cheap, and a suitable carrier due to its nonspherical shape in the nanofibrous form attractive to the pharmaceutical/biomedical industries. Additionally, nanocellulose has hydroxyl groups that might form a hydrogen bonding with spike glycoproteins and stabilize the ligand–receptor complex. For future opportunities, we believe in new attempts with the non‐toxic nanomaterials and more efforts in nanomaterials that have already been reported in non‐cytotoxicity levels as antiviral agents. Thus, trends in toxicology evaluation and safety tests of strategies reviewed can help fill the main gaps in the literature and overcome nanomaterials′ main challenges to health surveillance. Encapsulated nanosystems with v‐ATPase inhibition could be a promissive target therapeutic to overcome the challenge of antiviral drug resistance. Future directions can be identified in the opportunity to study the pharmacokinetics of phytochemicals delivered by nanomaterials cited to evaluate the effects in targeting, circulation time, and the ability to overcome biological barriers for drugs repurposing with more healthy options. Beyond that, the absence of attempts with nonspherical nanoparticles (e.g., filamentous shapes) could encourage new future efforts. We hope that our study's notes addressing the role of nanotechnology approaches with anti‐coronavirus properties can help researchers with insights to overcome the challenges associated with the SARS‐CoV‐2 virus control, given direction to develop novel antiviral therapies, and prevent future pandemics similar to the current COVID‐19.

## Conflict of Interest

The authors declare no conflict of interest.

## Author Contributions

The manuscript was written through all author's contributions: the systematic literature search and the studies revision were planned and performed by A.P.A.C. and C.A.C.J., and A.P.A.C. wrote the manuscript.
